# Real-world effectiveness of osteoporosis therapies for fracture reduction in post-menopausal women

**DOI:** 10.1007/s11657-018-0439-3

**Published:** 2018-03-21

**Authors:** Akeem A. Yusuf, Steven R. Cummings, Nelson B. Watts, Maurille Tepie Feudjo, J. Michael Sprafka, Jincheng Zhou, Haifeng Guo, Akhila Balasubramanian, Cyrus Cooper

**Affiliations:** 1 Center for Observational Research (CfOR), 1 Amgen Center Drive, MS 24-2-A, Thousand Oaks, CA 91320 USA; 2 Department of Medicine, University of California, San Francisco, CA USA; 3 Mercy Health Osteoporosis and Bone Health Services, Cincinnati, OH USA; 4 Amgen Inc., Uxbridge, UK; 5 Division of Biostatistics, School of Public Health, University of Minnesota, Minneapolis, MN USA; 6 Chronic Disease Research Group, Minneapolis, MN USA; 7 MRC Lifecourse Epidemiology Unit, University of Southampton, Southampton, UK

**Keywords:** Osteoporosis, Fracture risk reduction, Effectiveness, Anabolics, Antiresorptives

## Abstract

**Summary:**

Studies examining real-world effectiveness of osteoporosis therapies are beset by limitations due to confounding by indication. By evaluating longitudinal changes in fracture incidence, we demonstrated that osteoporosis therapies are effective in reducing fracture risk in real-world practice settings.

**Introduction:**

Osteoporosis therapies have been shown to reduce incidence of vertebral and non-vertebral fractures in placebo-controlled randomized clinical trials. However, information on the real-world effectiveness of these therapies is limited.

**Methods:**

We examined fracture risk reduction in older, post-menopausal women treated with osteoporosis therapies. Using Medicare claims, we identified 1,278,296 women age ≥ 65 years treated with zoledronic acid, oral bisphosphonates, denosumab, teriparatide, or raloxifene. Fracture incidence rates before and after treatment initiation were described to understand patients’ fracture risk profile, and fracture reduction effectiveness of each therapy was evaluated as a longitudinal change in incidence rates.

**Results:**

Fracture incidence rates increased during the period leading up to treatment initiation and were highest in the 3-month period most proximal to treatment initiation. Fracture incidence rates following treatment initiation were significantly lower than before treatment initiation. Compared with the 12-month pre-index period, there were reductions in clinical vertebral fractures for denosumab (45%; 95% confidence interval [CI] 39–51%), zoledronic acid (50%; 95% CI 47–52%), oral bisphosphonates (24%; 95% CI 22–26%), and teriparatide (72%; 95% CI 69–75%) during the subsequent 12 months. Relative to the first 3 months after initiation, clinical vertebral fractures were reduced for denosumab (51%; 95% CI 42–59%), zoledronic acid (25%; 95% CI 17–32%), oral bisphosphonates (23%; 95% CI 20–26%), and teriparatide (64%; 95% CI 58–69%) during the subsequent 12 months.

**Conclusion:**

In summary, reductions in fracture incidence over time were observed in cohorts of patients treated with osteoporosis therapies.

**Electronic supplementary material:**

The online version of this article (10.1007/s11657-018-0439-3) contains supplementary material, which is available to authorized users.

## Introduction

Osteoporosis is a disease characterized by compromised bone strength and increased risk of fractures. Prevalence of osteoporosis in the USA is estimated to increase to over 14 million people by 2020 [1]. Osteoporosis-related fractures are associated with increased morbidity, mortality, functional debility, health service utilization, health care costs, and loss of quality of life [2–5]. Prevention of osteoporosis-related fractures is the key goal of pharmacotherapy in osteoporosis patients.

Medications available for osteoporosis management include bisphosphonates, raloxifene, teriparatide, denosumab, calcitonin, and abaloparatide. While these agents have demonstrated increased bone mineral density and decreased fracture risk in placebo-controlled trials [6–9], there are limited data on their effectiveness in reducing fracture risk in real-world clinical practice settings. Sub-optimal adherence to medications, as well as heterogeneity in patient populations, could lead to differences in treatment effectiveness observed in clinical practice compared with randomized controlled trials.

Several observational studies have examined the effectiveness of osteoporosis medications using secondary data [10–12]. However, these studies are limited to a few agents and are subject to confounding by indication because of systematic differences in the fracture risk level of patients prescribed different medications in clinical practice settings. The objectives of this study include (1) examining the fracture risk profile of patients treated with osteoporosis medications by describing fracture incidence before and after treatment initiation and (2) examining longitudinal changes in fracture incidence as a measure of treatment effectiveness.

## Methods

### Methodological approach

Inherent differences in pre-treatment risk profiles in treated patients pose a serious challenge when evaluating treatment effectiveness in real-world settings. Evaluating longitudinal changes in fracture incidence using a within-user design can mitigate confounding in analysis of treatment effectiveness. By measuring the change in fracture incidence over time, each treatment group can serve as its own control and the confounding effect of time-invariant factors are minimized. In this study, we examined treatment effectiveness by assessing longitudinal change in fracture incidence. First, we compared fracture incidence during the 12 months before treatment initiation with that in the subsequent 12-month period. Second, we compared fracture incidence during the first 3 months on treatment with that in the subsequent 12-month period. This approach has been previously employed to assess fracture risk reduction in patients treated with bisphosphonates [13].

### Data and study population

The data for this study were from the US Centers for Medicare and Medicaid Services’ Chronic Condition Warehouse. Medicare is a US federal health insurance program that provides medical and pharmacy benefits to all citizens aged 65 years or older, as well as to individuals with certain disabilities or with end-stage renal disease. The study population was drawn from a data sample including 100% of Medicare beneficiaries aged 65 years or older with evidence of osteoporosis, indicated by at least one inpatient or outpatient record for osteoporosis (International Classification of Diseases, Ninth Revision, Clinical Modification [ICD-9-CM] diagnosis code 733.0X) between 2008 and 2011.

Women who were aged 65 years or older and treated with zoledronic acid, alendronate, risedronate, ibandronate, raloxifene, teriparatide, or denosumab between January 1, 2009 and June 30, 2012 were identified. The index date was the date of treatment initiation. Eligible patients were required to be alive and continuously enrolled in Medicare Parts A, B, and D (without participation in a health maintenance organization) for at least 12 months before and for 4 months after the treatment index date. Patients with evidence of malignant neoplasm or carcinoma (excluding non-melanoma skin cancer), those with Paget’s disease of bone, or chemotherapy or radiation therapy for cancer in the 12 months before or 3 months after the treatment index date, and those who switched from the assigned medication group to another within 3 months after the treatment index date were excluded.

A hierarchical approach was employed in assigning patients to one of five treatment groups (denosumab, zoledronic acid, oral bisphosphonates, raloxifene, or teriparatide) to maximize the cohort size for newer therapies. The designed hierarchy was based on the relative order of entry into the US marketplace of the above treatments, with late entry receiving higher priority.

### Outcomes

Incident fractures at any skeletal site other than skull, face, finger, or toe (defined as “any” fracture) were identified using a combination of relevant ICD-9-CM diagnosis codes and Healthcare Common Procedure Coding System (HCPCS) procedure codes. In addition, we separately identified fractures at the hip, vertebra, and wrist. The fracture identification algorithm was designed to capture hospitalized fractures and fractures diagnosed in outpatient settings that required surgical repair, or evidence of spine imaging (for some clinical vertebral fractures) [14, 15]. Sequential claims for fractures occurring at the same skeletal location had to be separated by 90 or more days, during which there was no fracture diagnosis code, in order to represent distinct fracture events.

### Covariate ascertainment

Patient demographic and clinical characteristics (including age, race, hospitalizations, fragility fracture, bone density testing, and comorbid conditions) were assessed at the time of treatment initiation and during the 12-month pre-index period. Baseline exposures to bone-altering medications, including glucocorticosteroids and osteoporosis medications other than the index medication, were also described (Table 1).

**Table 1:** Characteristic of female Medicare beneficiaries treated with osteoporosis therapies

	Denosumab	Zoledronic acid	Oral bisphosphonates	Raloxifene	Teriparatide
Sample size	34,622	124,857	997,686	100,521	20,610
Age^a^, mean (SD), year	78.7 (7.4)	77.0 (7.0)	78.2 (7.5)	77.9 (7.4)	78.5 (7.6)
Race (%)					
White	85.8	93.6	83.7	85.1	83.8
Black	2.4	2.4	4.5	3.0	2.3
Asian	6.6	1.4	5.4	7.3	4.6
Hispanic	2.9	1.5	4.0	2.3	7.2
Other	2.2	1.1	2.4	2.5	2.1
Index year^b^ (%)					
2009	0.0	43.8	80.6	87.9	4.0
2010	13.2	29.1	10.6	6.2	19.0
2011	54.8	20.8	6.8	4.2	18.6
2012	32.1	6.4	2.0	1.7	8.4
Census region (%)					
Northeast	12.5	14.3	20.4	21.6	15.5
Midwest	25.1	28.8	23.1	21.3	18.0
South	39.3	43.8	36.6	38.7	48.8
West	23.0	13.1	19.6	17.7	17.5
Comorbid conditions^c^ (%)					
Chronic kidney disease	14.7	6.2	7.4	7.2	11.7
Atherosclerotic heart disease	20.5	17.9	17.9	15.6	24.8
Congestive heart failure	11.7	8.5	10.1	8.2	16.1
Cerebrovascular disease	11.4	9.7	9.9	7.9	13.6
Dysrhythmia	19.4	16.3	15.9	13.2	20.6
Peripheral arterial disease	16.4	13.6	14.7	11.7	20.5
Other cardiac disease	18.0	15.3	14.2	13.0	19.6
Chronic obstructive pulmonary disease	20.7	20.3	17.1	15.4	26.9
Liver disease	1.3	1.1	1.0	0.9	1.5
Gastrointestinal disorders	4.3	4.1	2.9	3.1	4.9
Anemia	27.2	21.4	19.7	18.2	33.0
Diabetes	21.4	16.9	21.3	19.9	22.5
Thyroid disease	29.7	27.6	23.7	22.9	28.9
Rheumatoid arthritis	7.2	8.0	4.4	2.9	8.9
Ankylosing spondylitis	1.4	1.4	0.8	0.7	1.8
Osteoporosis	88.2	90.5	46.2	37.6	83.2
Osteoarthritis	34.8	32.6	28.4	26.1	41.9
Charlson comorbidity index^d^, mean (SD)	0.90 (1.3)	0.70 (1.1)	0.72 (1.2)	0.61 (1.1)	1.13 (1.5)
Hospitalization ^c^					
Any hospitalization (%)	22.3	21.9	20.3	17.0	34.9
Cumulative hospital days, mean (SD)	1.47 (4.7)	1.39 (4.5)	1.43 (5.3)	1.10 (4.3)	2.98 (7.3)
Patients with bone density test^c^ (%)	61.3	60.3	37.5	30.7	60.9
Patients with prior fracture ^c^ (%)	10.2	9.3	7.0	4.4	25.0
Patients with incident medication use^e^ (%)	34.9	30.8	65.9	79.6	56.7
Glucocorticoid use^c^ (%)	29.8	31.5	19.9	18.2	31.0

*SD* standard deviation

^a^On the index date

^b^Year of index date

^c^During the 12 months preceding the index date

^d^Computed from claims during the 12 months preceding the index date and based on Quan’s modification

^e^Defined as absence of claims for osteoporosis medications (including calcitonin) in the 6 months preceding the index date

### Statistical analysis

Fracture incidence (any, hip, clinical vertebral, and wrist) during the 12-month pre-index period (overall and in 3-month intervals) and during the post-index period was computed. Fracture reduction effectiveness was estimated as the change in fracture incidence using two approaches. First, change in fracture incidence was estimated between the 12-month pre-index period and the first 12 months following treatment initiation. Fracture reduction in the period immediately following treatment initiation is expected to be minimal and can serve as a baseline for estimating change in fracture incidence. Thus, for the second approach, change in fracture incidence was estimated between the first 3 months on treatment (early period) and the subsequent 12-month on-treatment period. For both approaches, patients were at risk for fracture outcomes from the index date until occurrence of a censoring event during the on-treatment period (death, discontinuation of Medicare enrolment, switching from index medication to a different study medication or calcitonin, diagnosis of cancer [excluding non-melanoma skin cancer] or Paget’s disease, chemotherapy or radiation therapy for cancer treatment). Change in fracture incidence was estimated as incidence rate ratios (with corresponding 95% confidence intervals) using generalized estimating equations with a Poisson link function.

In addition, stratified analysis was conducted for those with and without a history of osteoporosis medication use or prior fracture [16, 17].

## Results

### Cohort characteristics

**Fig. 1:**
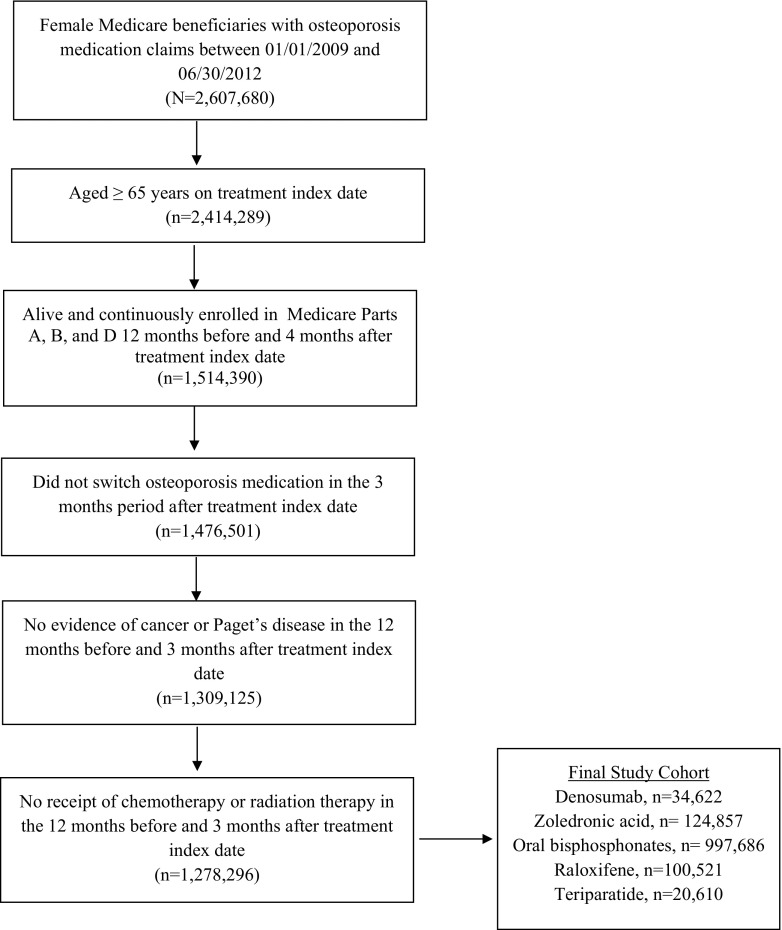
Cohort creation flow chart

After application of the inclusion and exclusion criteria, 34,622 denosumab, 124,857 zoledronic acid, 997,686 oral bisphosphonate, 100,521 raloxifene, and 20,610 teriparatide patients were included in the study cohort (Fig. 1). The average age of the study cohort was approximately 78 years. Teriparatide and raloxifene patients had the highest and lowest comorbidity burden, respectively. The average risk of fracture during the 1-year baseline period was highest among teriparatide patients (25.0%) and lowest among raloxifene patients (4.4%) (Table 1).

### Fracture incidence rates prior to treatment initiation

**Fig. 2:**
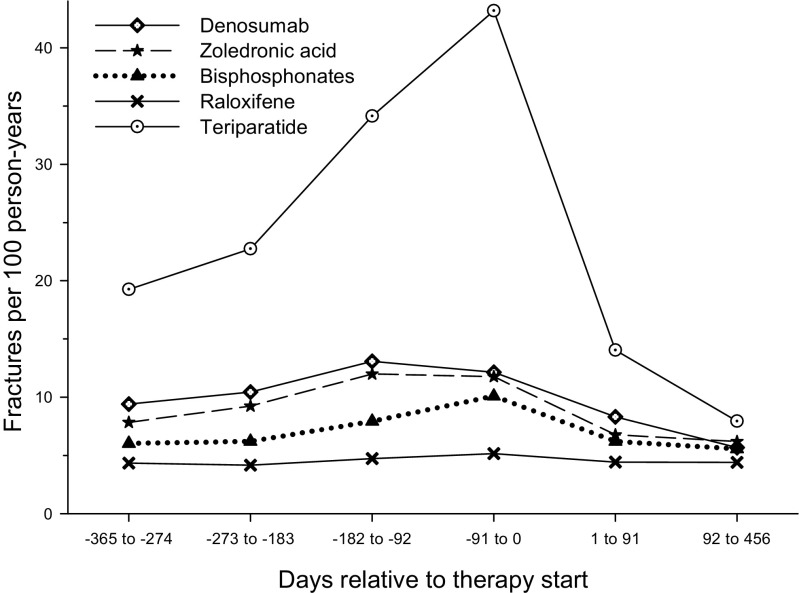
Pre-index and post-index fracture incidence rates among patients treated with osteoporosis medications (fractures were identified at any skeletal site other than skull, face, finger, or toe)

There was substantial variation in the magnitude and trajectory of fracture incidence rates among treatment groups in the 12-month pre-index period (Fig. 2). Fracture incidence rates were highest for teriparatide and lowest for raloxifene during each of the four 3-month intervals in the pre-index period. During the 3-month period leading up to treatment initiation, teriparatide patients had an eightfold higher risk of fracture compared with raloxifene patients. For all treatment groups, fracture incidence rates tended to increase during the pre-index period and then regress rather dramatically during the 3-month period following treatment initiation.

### Baseline fracture incidence

**Fig. 3:**
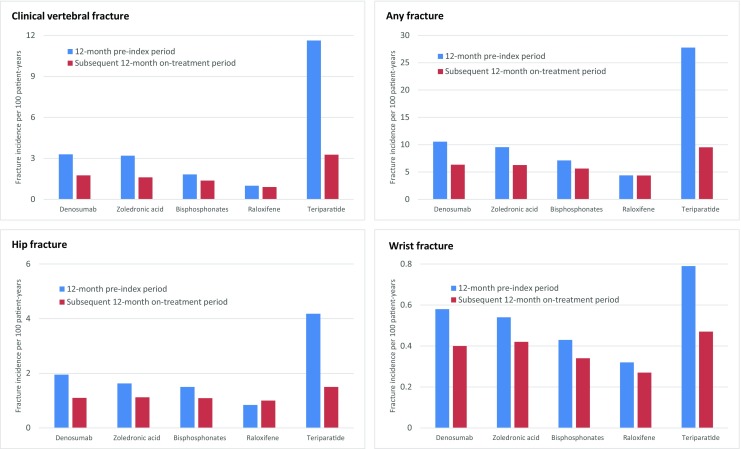
Change in fracture incidence rates between 12-month pre-index and subsequent on-treatment period. “Any” fracture is a composite endpoint of fractures at any skeletal site other than skull/face/finger/toe

**Fig. 4:**
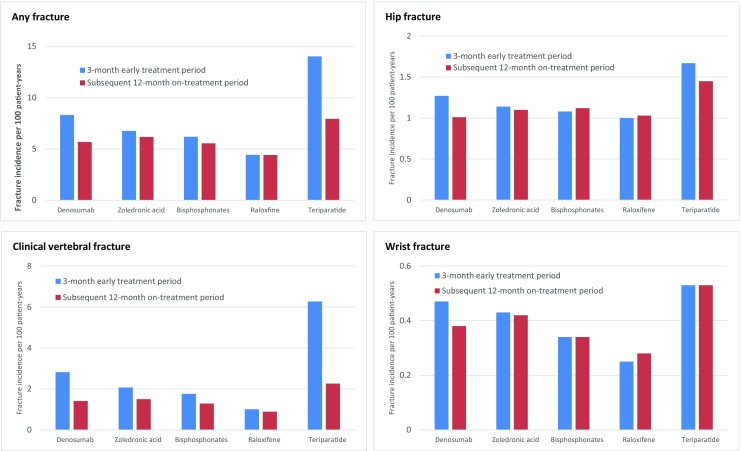
Change in fracture incidence rates between 3-month early treatment period and subsequent on-treatment period. “Any” fracture is a composite endpoint of fractures at any skeletal site other than skull/face/finger/toe

During the 12-month pre-index period as well as during the 3-month early treatment period, teriparatide-treated patients had the highest fracture incidence whereas raloxifene-treated patients had the lowest risk for any, hip, clinical vertebral, and wrist fracture (Figs. 3 and 4). Compared to rates among oral bisphosphonate patients, incidence rates of any fracture in teriparatide-treated patients were approximately fourfold greater during the 12-month pre-index period and double during the 3-month early treatment period (Table 2).

**Table 2:** Change in fracture incidence rates and corresponding incidence rate ratios

	Initial 3-month post-index versus subsequent period			12-month pre-index versus post-index		
	3-month early treatment period	Subsequent 12-month on-treatment period	IRR^a^ (95% CI)	12-month pre-index period	Subsequent 12-month on-treatment period	IRR^a^ (95% CI)
Any fracture^b^						
Denosumab	8.32	5.68	0.68 (0.62–0.75)	10.55	6.35	0.61 (0.57–0.64)
Zoledronic acid	6.77	6.19	0.96 (0.91–1.02)	9.55	6.28	0.65 (0.63–0.67)
Oral bisphosphonates	6.20	5.56	0.94 (0.92–0.96)	7.13	5.63	0.79 (0.78–0.80)
Raloxifene	4.43	4.41	1.08 (1.00–1.16)	4.39	4.36	0.99 (0.95–1.04)
Teriparatide	14.04	7.94	0.58 (0.53–0.65)	27.79	9.53	0.34 (0.32–0.36)
Hip						
Denosumab	1.27	1.01	0.80 (0.64–1.01)	1.95	1.10	0.56 (0.49–0.64)
Zoledronic acid	1.14	1.10	0.97 (0.86–1.09)	1.63	1.12	0.69 (0.64–0.74)
Oral bisphosphonates	1.08	1.12	1.05 (1.00–1.09)	1.50	1.09	0.73 (0.71–0.75)
Raloxifene	1.00	1.03	1.04 (0.90–1.19)	0.84	1.00	1.19 (1.09–1.31)
Teriparatide	1.67	1.45	0.87 (0.68–1.11)	4.18	1.50	0.36 (0.31–0.41)
Clinical vertebral						
Denosumab	2.82	1.41	0.49 (0.41–0.58)	3.29	1.75	0.55 (0.49–0.61)
Zoledronic acid	2.07	1.50	0.75 (0.68–0.83)	3.20	1.61	0.50 (0.48–0.53)
Oral bisphosphonates	1.76	1.29	0.77 (0.74–0.80)	1.82	1.37	0.76 (0.74–0.78)
Raloxifene	1.01	0.89	0.96 (0.82–1.13)	1.00	0.90	0.90 (0.82–0.99)
Teriparatide	6.27	2.26	0.36 (0.31–0.42)	11.63	3.27	0.28 (0.25–0.31)
Wrist						
Denosumab	0.47	0.38	0.83 (0.57–1.19)	0.58	0.40	0.70 (0.55–0.89)
Zoledronic acid	0.43	0.42	0.99 (0.82–1.20)	0.54	0.42	0.77 (0.69–0.87)
Oral bisphosphonates	0.34	0.34	1.02 (0.94–1.10)	0.43	0.34	0.78 (0.75–0.82)
Raloxifene	0.25	0.28	1.11 (0.84–1.47)	0.32	0.27	0.84 (0.71–0.99)
Teriparatide	0.53	0.53	1.03 (0.66–1.62)	0.79	0.47	0.60 (0.46–0.77)

*CI* confidence interval, *IRR* incidence rate ratio

^a^Separate Poisson models were estimated for any, hip, vertebral, and wrist fractures using generalized estimating equations

^b^A composite endpoint of fractures at any skeletal site other than skull/face/finger/toe

### Fracture incidence rates following treatment initiation

Compared with the 12-month pre-index period, incidence of any, hip, clinical vertebral, or wrist fracture was significantly lower in the subsequent 12-month on-treatment period among denosumab-, zoledronic acid-, oral bisphosphonate-, and teriparatide-treated patients. The incidence of clinical vertebral fractures was reduced with denosumab (45%; 95% confidence interval [CI] 39–51%), zoledronic acid (50%; 95% CI 47–52%), oral bisphosphonates (24%; 95% CI 22–26%), and teriparatide (72%; 95% CI 69–75%). Among raloxifene-treated patients the incidence of clinical vertebral fracture (10%; 95% CI 1–18%) and wrist fracture (16%; 95% CI 1–29%) was reduced significantly between the 12-month pre-index and 12-month post-index periods (Table 2, Fig. 3).

Compared with the 3-month early treatment period, the incidence of any fracture or clinical vertebral fracture was significantly lower in the subsequent 12-month on-treatment period among denosumab-, oral bisphosphonate-, and teriparatide-treated patients. In addition, zoledronic acid-treated patients had a significant reduction in the incidence of clinical vertebral fractures in the 12-month subsequent on-treatment period relative to the 3-month early treatment period. The incidence of clinical vertebral fractures was reduced with denosumab (51%; 95% CI 42–59%), zoledronic acid (25%; 95% CI 17–32%), oral bisphosphonates (23%; 95% CI 20–26%), and teriparatide (64%; 95% CI 58–69%) (Table 2, Fig. 4).

### Stratified and sensitivity analyses

In all the treatment cohorts, greater reductions in fracture incidence between the first 3 months on treatment and subsequent on-treatment periods were observed among treatment-naïve patients than among those with prior exposure to osteoporosis medications. Among patients with prior exposure to osteoporosis medications, only denosumab- and teriparatide-treated patients showed significant reductions in clinical vertebral fracture and any fracture endpoints. Similar reductions were not observed among previously treated patients in the oral bisphosphonate, zoledronic acid, or raloxifene cohorts (Supplemental Table 1). Across all treatment cohorts, the magnitude of reductions in fracture incidence during the on-treatment period was greater among patients with a history of prior fracture (Supplemental Table 2).

## Discussion

This study focused on the real-world effectiveness of osteoporosis therapies in a large, population-based cohort of post-menopausal women. Our results show substantial variation in fracture risk in the period preceding treatment initiation among patients treated with different osteoporosis medications. Significant reductions in hip, clinical vertebral, and wrist fractures among patients treated with denosumab, zoledronic acid, oral bisphosphonates, and teriparatide were observed following treatment initiation.

Evidence from randomized clinical trials and network meta-analyses has shown that osteoporosis medications are effective in reducing the incidence of vertebral and non-vertebral fractures in post-menopausal women [6–9, 18–21]. However, because of the differences in patient characteristics and non-adherence in the general population of treated patients, the demonstrated efficacy in clinical trials may not correlate with effectiveness in routine clinical settings. The most recent systematic review of the effectiveness of osteoporosis medications reported that bisphosphonates, denosumab, teriparatide, and raloxifene reduced clinical vertebral fracture risk relative to placebo [22]. Despite a shorter follow-up duration relative to registrational clinical trials, the findings of this study were largely consistent with evidence from randomized clinical trials of osteoporosis treatments in showing effective fracture incidence reduction.

The quarterly incidence analysis showed an increasing fracture risk in the period leading up to treatment initiation, and fracture incidence was highest in the 3-month period most proximal to therapy initiation for all treatment groups. This suggests that occurrence of fracture likely triggers the initiation of therapy in many patients. Given the very high pre-index fracture risk of teriparatide-treated patients relative to the other treatment groups, it appears that teriparatide is used as a rescue therapy in this population. In observational studies of treatment effectiveness, differences in risk profile and other pre-treatment characteristics can lead to biased effectiveness estimates. In this study, we employed an approach that can mitigate against bias by measuring changes in fracture incidence over time within the same user cohort. The trends in fracture incidence (as shown in Fig. 2) strongly suggest a regression to the mean effect that is differential by treatments. Between 12 months before and 3 months after treatment initiation, fracture risk for raloxifene users showed little variance whereas that for teriparatide users showed substantial variance. Thus, it is likely that the true treatment effect lies somewhere in between the estimates provided by the two analytic approaches. The most consistent treatment effects irrespective of analytic technique, fracture site, or treatment history were observed for denosumab and teriparatide. An alternative analysis that might be conducted in a future research investigation will be to assess the first 3 months after treatment initiation as a “wash-out” period and evaluate change in fracture rates between the pre-index period and the post-index period excluding the first 3 months.

The findings of this study showed substantial differences in the pre-treatment fracture risk across the different treatment options which are highly suggestive of confounding by indication. Thus, investigators should be aware of this issue and offer appropriate technique to minimize bias due to confounding by indication when they design studies to examine comparative effectiveness of osteoporosis therapies.

A few observational studies have examined longitudinal changes in fracture incidence among patients treated with osteoporosis medications by comparing fracture incidence in the first 3 months after start of treatment to the subsequent 12 months on treatment. Abelson et al. reported a vertebral fracture incidence reduction ranging from 31 to 57% among women treated with oral bisphosphonates [13]. Ferrari et al. estimated a hip fracture incidence reduction of 17% among women treated with risedronate, but found no change in fracture incidence among those treated with raloxifene [23]. Thomas et al. reported a 59 and 54% vertebral fracture incidence reduction among women treated with alendronate and risedronate, respectively [24]. Our fracture reduction estimates are lower than those reported in these studies and the explanation for this finding may be attributable to the different populations studied and secular trends, as our study population is older and may be at a higher risk of fractures and we analyzed more recent data.

The analysis comparing fracture rates in the 12 months pre-index period to the fracture rates in the post-index period showed substantial reduction in hip fracture risk among patients treated with denosumab, zoledronic acid, oral bisphosphonates, and teriparatide. However, there was minimal evidence of fracture risk reduction when fracture risk in the initial 3-month post-index was compared to the subsequent 12-month on-treatment period. While the exact reasons for the apparent lack of evidence for hip fracture reduction in the latter analysis are unclear, it is possible that the follow-up period was too short to identify a reduction in hip fracture rates.

Despite our approach of evaluating changes in fracture incidence over time within the same user cohort, threats to internal validity may be present. Fracture risk is highest immediately after a prior fracture and falls gradually over time [25, 26]; hence, it can be expected that for those individuals who had a prior fracture within the last few years before starting the index medication that fracture rates would have continue to fall over the first year of therapy even in the absence of therapy. Thus, bias due to regression to the mean likely exists for the comparison of pre-index and post-index fracture incidence.

Definitive identification of denosumab users in the Medicare population in the early period of its introduction is challenging because of the use of non-specific codes for billing. Although denosumab users were identified using a validated algorithm [27], the possibility of exposure misclassification cannot be ruled out. The hierarchical treatment assignment approach may have created some bias, possibly via misclassification, and patients assigned to treatments that received higher priority were more likely to be at a more advanced stage of their disease course. The direction of this potential bias is unclear. In addition, morphometric vertebral fractures that represent the majority of vertebral fractures in clinical trials cannot be identified in claim databases. Finally, Medicare Part D beneficiaries who do not receive the low-income subsidy are subject to cost sharing for prescriptions filled through Medicare Part D and they may fall into the coverage gap [28]. Hence, they may obtain some prescriptions outside of the Medicare Part D system and their osteoporosis medication use may thus be misclassified.

In summary, this large population-based study showed that clinical fracture incidence decreased after initiation of osteoporosis therapies.

## Electronic supplementary material

ESM 1 (DOCX 41 kb)

## References

[1] American Academy of Orthopaedic Surgeons. Osteoporosis/Bone Health in Adults as a National Public Health Priority. Position Statement. http://www.aaos.org/about/papers/position/1113.asp. Accessed 3 April, 2017

[2] Mauck KF, Clarke BL (2006). Diagnosis, screening, prevention, and treatment of osteoporosis. Mayo Clin Proc.

[3] Rousculp MD, Long SR, Wang S, Schoenfeld MJ, Meadows ES (2007). Economic burden of osteoporosis-related fractures in Medicaid. Value Health.

[4] Liu SK, Munson JC, Bell JE, Zaha RL, Mecchella JN, Tosteson ANA, Morden NE (2013). Quality of osteoporosis care of older Medicare recipients with fragility fractures: 2006 to 2010. J Am Geriatr Soc.

[5] Tajeu GS, Delzell E, Smith W (2014). Death, debility, and destitution following hip fracture. J Gerontol A Biol Sci Med Sci.

[6] Bilezikian JP (2009). Efficacy of bisphosphonates in reducing fracture risk in postmenopausal osteoporosis. Am J Med.

[7] Cummings SR, San Martin J, McClung MR, Siris ES, Eastell R, Reid IR, Delmas P, Zoog HB, Austin M, Wang A, Kutilek S, Adami S, Zanchetta J, Libanati C, Siddhanti S, Christiansen C, FREEDOM Trial (2009). Denosumab for prevention of fractures in postmenopausal women with osteoporosis. N Engl J Med.

[8] Black DM, Delmas PD, Eastell R, Reid IR, Boonen S, Cauley JA, Cosman F, Lakatos P, Leung PC, Man Z, Mautalen C, Mesenbrink P, Hu H, Caminis J, Tong K, Rosario-Jansen T, Krasnow J, Hue TF, Sellmeyer D, Eriksen EF, Cummings SR, HORIZON Pivotal Fracture Trial (2007). Once-yearly zoledronic acid for treatment of postmenopausal osteoporosis. N Engl J Med.

[9] Neer RM, Arnaud CD, Zanchetta JR, Prince R, Gaich GA, Reginster JY, Hodsman AB, Eriksen EF, Ish-Shalom S, Genant HK, Wang O, Mellström D, Oefjord ES, Marcinowska-Suchowierska E, Salmi J, Mulder H, Halse J, Sawicki AZ, Mitlak BH (2001). Effect of parathyroid hormone (1-34) on fractures and bone mineral density in postmenopausal women with osteoporosis. N Engl J Med.

[10] Cadarette SM, Katz JN, Brookhart MA, Sturmer T, Stedman MR, Solomon DH (2008). Relative effectiveness of osteoporosis drugs for preventing nonvertebral fracture. Ann Intern Med.

[11] Lin TC, Yang CY, Yang YH, Lin SJ (2013). Comparative effectiveness of osteoporosis drugs in preventing secondary nonvertebral fractures in Taiwanese women. J Clin Endocrinol Metab.

[12] Yun H, Delzell E, Saag KG, Kilgore ML, Morrisey MA, Muntner P, Matthews R, Guo L, Wright N, Smith W, Colón-Emeric C, O'Connor CM, Lyles KW, Curtis JR (2015). Fractures and mortality in relation to different osteoporosis treatments. Clin Exp Rheumatol.

[13] Abelson A, Ringe JD, Gold DT, Lange JL, Thomas T (2010). Longitudinal change in clinical fracture incidence after initiation of bisphosphonates. Osteoporos Int.

[14] Ray WA, Griffin MR, Fought RL, Adams ML (1992). Identification of fractures from computerized Medicare files. J Clin Epidemiol.

[15] Curtis JR, Mudano AS, Solomon DH, Xi J, Melton ME, Saag KG (2009). Identification and validation of vertebral compression fractures using administrative claims data. Med Care.

[16] Prieto-Alhambra D, Pagès-Castellà A, Wallace G, Javaid MK, Judge A, Nogués X, Arden NK, Cooper C, Diez-Perez A (2014). Predictors of fracture while on treatment with oral bisphosphonates: a population-based cohort study. J Bone Miner Res.

[17] Taylor AJ, Gary LC, Arora T, Becker DJ, Curtis JR, Kilgore ML, Morrisey MA, Saag KG, Matthews R, Yun H, Smith W, Delzell E (2011). Clinical and demographic factors associated with fractures among older Americans. Osteoporos Int.

[18] Hopkins RB, Goeree R, Pullenayegum E, Adachi JD, Papaioannou A, Xie F, Thabane L (2011). The relative efficacy of nine osteoporosis medications for reducing the rate of fractures in post-menopausal women. BMC Musculoskelet Disord.

[19] Murad MH, Drake MT, Mullan RJ, Mauck KF, Stuart LM, Lane MA, Abu Elnour NO, Erwin PJ, Hazem A, Puhan MA, Li T, Montori VM (2012). Clinical review. Comp Eff Drug Treat Prev Fragility Fractures.

[20] Freemantle N, Cooper C, Diez-Perez A, Gitlin M, Radcliffe H, Shepherd S, Roux C (2013). Results of indirect and mixed treatment comparison of fracture efficacy for osteoporosis treatments: a meta-analysis. Osteoporos Int.

[21] Migliore A, Broccoli S, Massafra U, Cassol M, Frediani B (2013). Ranking antireabsorptive agents to prevent vertebral fractures in postmenopausal osteoporosis by mixed treatment comparison meta-analysis. Eur Rev Med Pharmacol Sci.

[22] Crandall CJ, Newberry SJ, Diamant A, Lim YW, Gellad WF, Booth MJ, Motala A, Shekelle PG (2014). Comparative effectiveness of pharmacologic treatments to prevent fractures. Ann Intern Med.

[23] Ferrari S, Nakamura T, Hagino H, Fujiwara S, Lange JL, Watts NB (2011). Longitudinal change in hip fracture incidence after starting risedronate or raloxifene: an observational study. J Bone Miner Metab.

[24] Thomas T, Horlait S, Ringe JD, Abelson A, Gold DT, Atlan P, Lange JL (2013). Oral bisphosphonates reduce the risk of clinical fractures in glucocorticoid-induced osteoporosis in clinical practice. Osteoporos Int.

[25] van Geel TA, Huntjens KM, van den Bergh JP, Dinant GJ, Geusens PP (2010). Timing of subsequent fractures after an initial fracture. Curr Osteoporos Rep.

[26] Johnell O, Kanis JA, Odén A, Sernbo I, Redlund-Johnell I, Petterson C, De Laet C, Jönsson B (2004). Fracture risk following an osteoporotic fracture. Osteoporos Int.

[27] Hoffman V, Xue F, Gardstein B, Skerry K, Critchlow CW, Enger C (2014). Development and evaluation of an algorithm to identify users of Prolia(®) during the early postmarketing period using health insurance claims data. Pharmacoepidemiol Drug Saf.

[28] Doherty RB (2004). Assessing the new Medicare prescription drug law. Ann Intern Med.

