# Supplementary material for: Real-world effectiveness of osteoporosis therapies for fracture reduction in post-menopausal women
Source: Arch Osteoporos. 2018 Mar 21;13(1):33. doi: 10.1007/s11657-018-0439-3 (PMC5862911; doi:10.1007/s11657-018-0439-3)
Supplement: Supplementary file 1 — (DOCX 41 kb) [file 11657_2018_439_MOESM1_ESM.docx]

Supplemental Table 1. Fracture Incidence Rate, Rate Ratios and Corresponding 95% Confidence Intervals Stratified by Baseline Use of Other Osteoporosis Therapies

|  | Had baseline use of osteoporosis treatments | | | | Had no baseline use of osteoporosis treatments | | | |
| --- | --- | --- | --- | --- | --- | --- | --- | --- |
|  | Incidence rate/100 patient-years | | Rate ratios | | Incidence rate/100 patient-years | | Rate ratios | |
|  | 3-month early treatment period | Subsequent 12-month on-treatment period | IRR | 95% CI | 3-month early treatment period | Subsequent 12-month on-treatment period | IRR | 95% CI |
| Any fracture |  |  |  |  |  |  |  |  |
| Denosumab | 8.01 | 5.20 | 0.65 | 0.55–0.77 | 8.49 | 5.94 | 0.70 | 0.62–0.79 |
| Zoledronic acid | 6.18 | 6.33 | 1.08 | 0.98–1.19 | 7.04 | 6.13 | 0.92 | 0.86–0.98 |
| Oral bisphosphonates | 5.35 | 5.40 | 1.06 | 1.04–1.09 | 7.85 | 5.87 | 0.78 | 0.76–0.80 |
| Raloxifene | 4.08 | 4.37 | 1.15 | 1.06–1.26 | 5.77 | 4.58 | 0.86 | 0.74–1.00 |
| Teriparatide | 12.06 | 7.48 | 0.65 | 0.56–0.76 | 16.64 | 8.54 | 0.52 | 0.46–0.60 |
| Hip |  |  |  |  |  |  |  |  |
| Denosumab | 1.40 | 0.82 | 0.58 | 0.40–0.85 | 1.20 | 1.11 | 0.94 | 0.71–1.25 |
| Zoledronic acid | 0.94 | 1.04 | 1.12 | 0.88–1.42 | 1.23 | 1.13 | 0.92 | 0.80–1.06 |
| Oral bisphosphonates | 0.95 | 1.07 | 1.12 | 1.06–1.19 | 1.31 | 1.23 | 0.94 | 0.88–1.01 |
| Raloxifene | 0.94 | 1.03 | 1.10 | 0.94–1.29 | 1.22 | 1.02 | 0.84 | 0.63–1.12 |
| Teriparatide | 1.14 | 1.38 | 1.21 | 0.83–1.77 | 2.36 | 1.54 | 0.65 | 0.47–0.90 |
| Clinical vertebral |  |  |  |  |  |  |  |  |
| Denosumab | 2.34 | 1.22 | 0.51 | 0.38–0.70 | 3.07 | 1.51 | 0.47 | 0.38–0.59 |
| Zoledronic acid | 1.83 | 1.63 | 0.92 | 0.77–1.10 | 2.17 | 1.44 | 0.68 | 0.61–0.77 |
| Oral bisphosphonates | 1.31 | 1.25 | 1.01 | 0.96–1.06 | 2.63 | 1.37 | 0.54 | 0.51–0.57 |
| Raloxifene | 0.88 | 0.88 | 1.10 | 0.91–1.34 | 1.49 | 0.92 | 0.65 | 0.48–0.89 |
| Teriparatide | 5.67 | 2.17 | 0.38 | 0.31–0.48 | 7.04 | 2.37 | 0.34 | 0.27–0.43 |
| Wrist |  |  |  |  |  |  |  |  |
| Denosumab | 0.43 | 0.41 | 0.96 | 0.51–1.80 | 0.48 | 0.37 | 0.76 | 0.48–1.20 |
| Zoledronic acid | 0.46 | 0.45 | 0.98 | 0.70–1.36 | 0.41 | 0.41 | 1.00 | 0.79–1.26 |
| Oral bisphosphonates | 0.32 | 0.33 | 1.04 | 0.95–1.15 | 0.37 | 0.36 | 0.98 | 0.86–1.11 |
| Raloxifene | 0.24 | 0.26 | 1.11 | 0.81–1.52 | 0.31 | 0.35 | 1.14 | 0.65–2.01 |
| Teriparatide | 0.59 | 0.52 | 0.88 | 0.51–1.53 | * | 0.54 | 1.27 | 0.59–2.76 |

Abbreviations: CI, confidence interval; IRR, incidence rate ratio. *Value suppressed because fracture count is less than 11.

Supplemental Table 2. Fracture Incidence Rate, Rate Ratios and Corresponding 95% Confidence Intervals Stratified by Baseline Fracture History

|  | No prior fracture | | | | Prior fracture history | | | |
| --- | --- | --- | --- | --- | --- | --- | --- | --- |
|  | Incidence rate/100 patient-years | | Rate ratios | | Incidence rate/100 patient-years | | Rate ratios | |
|  | 3-month early treatment period | Subsequent 12-month on-treatment period | IRR | 95% CI | 3-month early treatment period | Subsequent 12-month on-treatment period | IRR | 95% CI |
| Any fracture |  |  |  |  |  |  |  |  |
| Denosumab | 6.80 | 4.83 | 0.71 | 0.64–0.79 | 22.07 | 13.61 | 0.61 | 0.51–0.74 |
| Zoledronic acid | 5.47 | 5.39 | 1.03 | 0.97–1.10 | 19.75 | 14.68 | 0.78 | 0.70–0.87 |
| Oral bisphosphonates | 5.28 | 4.98 | 0.98 | 0.96–1.01 | 18.73 | 13.88 | 0.79 | 0.75–0.82 |
| Raloxifene | 3.89 | 4.02 | 1.11 | 1.03–1.21 | 16.44 | 13.65 | 0.88 | 0.73–1.07 |
| Teriparatide | 10.36 | 6.06 | 0.60 | 0.52–0.68 | 25.26 | 13.84 | 0.57 | 0.49–0.66 |
| Hip |  |  |  |  |  |  |  |  |
| Denosumab | 1.10 | 0.92 | 0.84 | 0.65–1.09 | 2.78 | 1.86 | 0.67 | 0.41–1.09 |
| Zoledronic acid | 1.01 | 0.98 | 0.98 | 0.86–1.12 | 2.45 | 2.33 | 0.94 | 0.73–1.23 |
| Oral bisphosphonates | 0.96 | 1.03 | 1.08 | 1.03–1.13 | 2.61 | 2.37 | 0.91 | 0.82–1.01 |
| Raloxifene | 0.92 | 0.94 | 1.04 | 0.89–1.20 | 2.78 | 2.97 | 1.06 | 0.71–1.58 |
| Teriparatide | 1.52 | 1.25 | 0.82 | 0.61–1.11 | 2.13 | 2.06 | 0.97 | 0.63–1.50 |
| Clinical vertebral |  |  |  |  |  |  |  |  |
| Denosumab | 2.13 | 1.16 | 0.53 | 0.43–0.65 | 9.01 | 3.71 | 0.40 | 0.30–0.55 |
| Zoledronic acid | 1.53 | 1.22 | 0.82 | 0.73–0.92 | 7.38 | 4.37 | 0.61 | 0.51–0.73 |
| Oral bisphosphonates | 1.42 | 1.10 | 0.81 | 0.78–0.85 | 6.38 | 3.94 | 0.65 | 0.60–0.70 |
| Raloxifene | 0.82 | 0.78 | 1.06 | 0.88–1.27 | 5.03 | 3.32 | 0.67 | 0.48–0.95 |
| Teriparatide | 4.60 | 1.54 | 0.33 | 0.27–0.41 | 11.34 | 4.47 | 0.41 | 0.32–0.52 |
| Wrist |  |  |  |  |  |  |  |  |
| Denosumab | 0.47 | 0.36 | 0.78 | 0.53–1.16 | * | 0.56 | 1.22 | 0.40–3.71 |
| Zoledronic acid | 0.39 | 0.40 | 1.01 | 0.82–1.24 | 0.73 | 0.66 | 0.92 | 0.56–1.51 |
| Oral bisphosphonates | 0.32 | 0.33 | 1.03 | 0.95–1.12 | 0.59 | 0.56 | 0.94 | 0.75–1.17 |
| Raloxifene | 0.24 | 0.27 | 1.13 | 0.85–1.51 | * | 0.44 | 0.95 | 0.35–2.58 |
| Teriparatide | 0.55 | 0.49 | 0.92 | 0.55–1.56 | * | 0.64 | 1.35 | 0.56–3.28 |

Abbreviations: CI, confidence interval; IRR, incidence rate ratio. *Value suppressed because fracture count is less than 11.

Supplemental Table 3. ICD-9-CM codes for comorbid conditions

| Comorbid conditions | ICD-9-CM codes |
| --- | --- |
| Chronic kidney disease | 016.0, 095.4, 189.0, 189.9, 223.0, 236.91,250.4, 271.4, 274.1, 283.11, 403.X1,404.X2, 404.X3, 440.1, 442.1, 447.3,572.4, 580-588, 591, 642.1, 646.2,753.12-753.17, 753.19, 753.2, and 794.4 |
| Atherosclerotic heart disease | 410-414, V45.18, and V45.82 |
| Congestive heart failure | 398.91, 422.xx, 425.xx, 428.xx, 402.x1,404.x1, 404.x3, V42.1 |
| Cerebrovascular disease | 430-438 |
| Dysrhythmia | 426.xx – 427.xx, V45.0, and V53.3 |
| Peripheral arterial disease | 440-444; 447; 451-453; 557 |
| Other cardiac disease | 420.xx – 421.xx, 423.xx – 424.xx, 429.xx, |
| Chronic obstructive pulmonary disease | 785.0 – 785.3, V42.2, and V43.3 |
| Liver disease | 491.xx – 494.xx, 496.xx, 510.xx |
| Gastrointestinal disorders | 570.xx, 571.xx, 572.1, 572.4, 573.1 – |
| Anemia | 280-285 |
| Diabetes | 250.xx, 357.2, 362.0x, 366.41 |
| Thyroid disease | 240.x-246.x |
| Rheumatoid arthritis | 714.x |
| Ankylosing spondylitis | 720.xx |
| Osteoarthritis | 715.xx |
